# Fracture prevalence during an unusual period of snow and ice in the Netherlands

**DOI:** 10.1186/1865-1380-7-17

**Published:** 2014-04-29

**Authors:** Crispijn L van den Brand, M Christien van der Linden, Naomi van der Linden, Steven J Rhemrev

**Affiliations:** 1 Emergency Department, Medical Centre Haaglanden, P.O. box 432, 2501 CK The Hague, The Netherlands; 2 Institute for Medical Technology Assessment, Erasmus University Rotterdam, P.O. box 1738, 3000 DR Rotterdam, The Netherlands; 3 Department of Trauma Surgery, Medical Centre Haaglanden, P.O. box 432, 2501 CK The Hague, The Netherlands

**Keywords:** Emergency medicine, Fractures, Prevention, Weather

## Abstract

**Background:**

The objective of the current study was to assess the effect of an unusual 10-day snow and ice period on the prevalence of fractures in an emergency department (ED) in the Netherlands. Furthermore, patients with fractures during the snow and ice period were compared to those in the control period with respect to gender, age, location of accident, length of stay, disposition, and anatomical site of the injury.

**Methods:**

Fracture prevalence during a 10-day study period with snow and ice (January 14, 2013 until January 23, 2013) was compared to a similar 10-day control period without snow or ice (January 16, 2012 until January 25, 2012). The records of all patients with a fracture were manually selected. Besides this, basic demographics, type of fracture, and location of the accident (inside or outside) were compared.

**Results:**

A total of 1,785 patients visited the ED during the study period and 1,974 during the control period. A fracture was found in 224 patients during the study period and in 109 patients during the control period (*P* <0.01). More fractures sustained outside account for this difference. No differences were found in gender, mean age, and length of ED stay. However, during the snow and ice period the percentage of fractures in the middle-aged (31–60 yrs) was significantly higher than in the control period (*P* <0.01).

**Conclusions:**

The number of fractures sustained more than doubled during a period with snow and ice as compared to the control period. In contrast to other studies outside the Netherlands, not the elderly, but the middle-aged were most affected by the slippery conditions.

## Background

Falls are a leading cause for emergency department (ED) visits [1]. During winter time, periods with snow and ice are associated with increased injuries [2,3]. In January 2013, a winter storm passed through the Netherlands, a country not commonly used to long periods of snow and ice. Temperatures dropped below freezing for days and streets and pavements were covered with snow and ice. Many major roads in the towns were salted, but most pavements and bicycle paths were left untreated with slippery and uneven surfaces dangerous for walking and cycling. National newspapers headlined ‘*Emergency departments busier than normal*’ [4], based on anecdotes of the ED nurses and doctors. The true effect of a snow and ice period in the Netherlands is not known.

Several studies have examined the effect of a snow and ice period on the frequency and type of injury [5-8], risk factors [5,9,10], aetiology [11], preventive measures [12-14], workload at an orthopaedic trauma-unit [15], and costs [15]. During a snow and ice period more injuries occur, leading to more clinical admissions and higher costs [15]. Preventive measures include advising people to go out only if necessary, instant cleaning of pavements, and anti-skid devices [2,6,12-14].

The aim of our study was to assess the effect of a 10-day snow and ice period on the number of fractures in a country not used to long periods of winter conditions. Furthermore, we assessed some epidemiological factors related to fractures incurred during a snow and ice period and during a control period. Our hypothesis was that the number of fractures sustained would increase during a period of snow and ice as compared with the control period.

## Methods

An observational, cross-sectional study was performed at two ED locations of a hospital in the west of the Netherlands: a level one trauma centre with 52,000 ED patient visits per year, and a level three community location with 24,000 ED patient visits per year.

The snow and ice period was from Monday, January 14^th^, 2013 until Wednesday, January 23^rd^, 2013. This snow and ice period was compared to a similar period (Monday, January 16^th^, 2012 until Wednesday, January 25^th^, 2012) in the previous year.

Details of weather conditions for the two time periods were gathered from a weather station in the Hague. Table 1 shows these details.

**Table 1:** Weather conditions during the two time periods (Weather Station, The Hague)

	**Snow and ice period**	**Control period**
	**(Jan 14**^**th**^**–Jan 23**^**rd **^**2013)**	**(Jan 16**^**th**^**–Jan 25**^**th **^**2012)**
Lowest daily mean temperature	−5.2°C (Jan 17^th^)	1.9°C (Jan 16^th^)
Highest daily mean temperature	−1.3°C (Jan 21^st^)	8.2°C (Jan 21^st^)
Lowest daily minimum temperature	−8.6°C (Jan 17^th^)	−0.4°C (Jan 16^th^)
Highest daily minimum temperature	−2.8°C (Jan 15^th^)	6.3°C (Jan 22^nd^)
Lowest daily maximum temperature	−3.6°C (Jan 17^th^)	5.4°C (Jan 16^th^)
Highest daily maximum temperature	3.0°C (Jan 16^th^)	10.3°C (Jan 22)

The electronic records of all patients with a diagnosed fracture were manually selected from all records of the patients who visited the ED during the 20 days (10-day snow and ice period and 10-day control period). The records of all patients who attended the ED during the 20 days were manually screened and all patients with clinically diagnosed and radiologically confirmed fractures were included. Patients with fractures sustained in the days before the snow and ice period and control period, who visited the ED during the snow and ice period and control period, were excluded.

Patients with a fracture who visited the ED during the snow and ice period were compared with patients with a fracture who visited the ED during the control period, with respect to gender, age, location of accident (outside or inside), length of stay (departure time minus registration time), disposition (outpatient clinic or admission to an inpatient unit), and anatomical site of the injury (head, arm, forearm and wrist, hand, spine and chest, hip, leg, ankle, and foot). Furthermore, we performed the same analyses for the patients with a fracture sustained outside.

### Analyses

Differences between patients during the snow and ice period and patients during the control period, with regards to gender, age, location of accident, length of stay, disposition, and anatomical site of the injury, were analysed using the *χ*^2^ test (categorical variables) and the Students *t*-test (continuous variables) equality of variances was tested with Levene’s test. In the absence of equality of variances the Mann–Whitney test was used. The same tests were used to assess differences between patients during the snow and ice period and the control period in gender, age, gender, location of accident, length of stay, disposition, and anatomical site of the injury between patients who fell outside their home. Where applicable, the relative risk (RR) was calculated; the hospital catchment population without fracture was used as patients with negative outcome (approximately 250,000). PASW (Predictive Analytics Soft Ware, version 20) was used. A *P* <0.05 was considered to be significant.

The study was registered and approved by the regional medical research ethics committee (METC ZWH) under number 13–053.

## Results

During the 20 days, 3,759 patient visits were registered at the two EDs; 1,785 ED visits during the snow and ice period, and 1,974 during the control period. A fracture was found in 332 patients; in 224 patients (12.5%) during the snow and ice period, and in 109 patients (5.5%) during the control period. For the entire hospital catchment population of 250,000 the RR for presenting to the ED with a fracture during the snow and ice period compared to the control period is 2.06 (95% CI, 1.63–2.58).

Table 2 shows patient and visit characteristics of patients with fractures who registered at the EDs during the snow and ice period and the control period. No differences were found in gender, age, and length of stay. We also did not find any differences in percentage of patients aged over 60 presenting with a fracture between the two periods. However, during the snow and ice period there were relatively less patients aged 0–30 years, and relatively more patients aged 31–60 years who presented with a fracture, compared to the control period.

**Table 2:** Patient and visit characteristics during the snow and ice period and during the control period

**Patients sustaining a fracture, visiting the emergency department (n = 333)**	**Snow and ice period (n = 224)**	**Control period (n = 109)**	***P *** **value**
Gender (n,%)			
Male	96 (42.9)	57 (52.3)	0.11
Female	128 (57.1)	52 (47.7)	
Age (mean, SD*)†	47.2 (21.7)	43.3 (26.6)	0.15
Age categories (n,%)			
0–15 years	28 (12.5)	24 (22.0)	0.03
16–30 years	26 (11.6)	24 (22.0)	0.01
31–60 years	107 (47.8)	28 (25.7)	<0.01
>60 years	63 (28.1)	33 (30.3)	0.68
Location (n,%)			
Level one trauma centre	135 (60.3)	65 (59.6)	0.91
Level three trauma centre	89 (39.7)	44 (40.4)	
Length of stay in minutes (mean, SD)‡	146 (87)	134 (86)	0.25
Disposition (n,%)			
Admission	31 (13.8)	20 (18.3)	0.28
Outpatient clinic	193 (86.2)	89 (81.7)	
Location of accident (n,%)			
Outside	153 (68.3)	42 (38.5)	<0.01
Inside	46 (20.5)	54 (49.5)	<0.01
Unknown	25 (11.2)	13 (11.9)	0.86

*SD = standard deviation.

†Levene’s test for equality of variances <0.01, Mann–Whitney test was used to compare groups.

‡Levene’s test for equality of variances 0.71, Student’s *t*-test was used to compare groups.

During the snow and ice period, 153 patients presented to the ED with a fracture as a result of a trauma outside; during the control period this number was 42 (RR, 3.64; 95% CI, 2.59–5.12). There was no difference in the number of fractures sustained inside between the two periods (RR, 0.85; 95% CI, 0.58–1.26). Assessing individual patient records showed that in 122 cases during the snow and ice period the fracture was probably a result of the weather conditions, in 37 cases this relationship was uncertain, and in 65 cases a relationship was unlikely.

During the snow and ice period 31 patients were admitted because of fractures, during the control period this number was 20, this difference was not significant (RR, 1.55; 95% CI, 0.88–2.72).

When focussing on anatomical location of fractures sustained during the snow and ice period (Table 3), there was a sharp increase in fractures happening when patients fell on the outstretched arm (forearm and hand fractures) and an increase in fractures as a result of ankle injury (ankle and foot fractures) was observed.

**Table 3:** Anatomic localization of fractures during the snow and ice period and during the control period

	Snow and ice period (n = 226)*	Control period (n = 112)*	**RR (95% CI)**
Head	4	1	4.00 (0.45–35.79)
Upper arm	21	14	1.50 (0.76–2.95)
Forearm	73	25	2.92 (1.85–4.60)
Hand	53	30	1.77 (1.13–2.76)
Chest and spine	14	10	1.40 (0.62–3.15)
Hip	12	10	1.20 (0.52–2.78)
Knee/leg	9	7	1.29 (0.48–3.45)
Ankle/foot	40	15	2.67 (1.47–4.83)

*Some patients had more than one fracture.

## Discussion

The main findings of our study are that the percentage of patients with fractures sustained during the snow and ice period more than doubled compared to the control period. The largest increase in fracture prevalence was seen in the 31–60 years age group. We found no increase in total number of ED patients, neither in the length of stay or in hospital admissions. The number of fractures sustained outside more than tripled while the number of fractures sustained inside did not increase during the period with snow and ice. Arm and ankle fractures were the most common.

The observed increase in absolute and relative fracture prevalence during a period with snow and ice compares well with other studies. A Welsh study found a 2.85 higher risk for patients to sustain a fracture during a snow and ice period than during a control period [2].

During the study period of 10 days of snow and ice, an estimated 122 patients with fractures as a direct result of weather conditions visited the two EDs. Although this is an alarming number, the actual snow and ice related injury is probably much higher, since only patients with fractures were included in our study. During a period with snow and ice many patients present at the ED with other injuries such as concussions, distortions, wounds, and dislocations. A Swedish study reports that approximately 50% of snow- and ice-related injuries at the ED are fractures [9].

To our surprise, length of stay of patients with fractures during the snow and ice period was not significantly longer than the length of stay of patients with fractures during the control period (149 vs. 134 minutes, *P* = 0.18). This could be explained by the fact that, in contrast to general belief, the ED was not busier during the period with snow and ice (1,785 vs. 1,974 patients) [4]. Possibly other, and not snow- and ice-related illnesses and injuries, occurred less often. Another explanation could be that during a period with snow and ice the threshold for visiting the ED is higher and people tend to stay at home with minor complaints. Other studies have mixed findings, while one study did report an increase in overall ED workload during a period with snow and ice [2], another large study supports our findings that the overall ED workload did not increase [3].

Other studies show a gradual increase in snow- and ice-related fracture risk with age and the elderly were most often affected [6,8]. In our study, in contrast, it was not the elderly but the middle-aged who were most affected by the winter weather conditions. This is interesting, also because the majority of the economically active population is in this age group [16] and therefore fractures in this age group result in an enormous economic burden for society. We presume this difference could possibly be explained because in a country not used to long periods of snow and ice, such as the Netherlands, the elderly tend to stay inside as much as possible. The middle-aged (31–60 years old), in contrast, have to go to work and are recreationally active. The same trend in fracture prevalence in different age groups was found in a Welsh study, another country not used to long periods of snow and ice [2]. Bicycling is a popular mode for commuter traffic in the Netherlands and ice-skating is a popular recreational activity [17]. Both ice-skating and bicycling on slippery roads are known risk factors for fractures and other injuries [18,19].

The catchment population of the hospitals in our study is about 250,000 [20]. Presuming our data can be extrapolated to the rest of the country would mean an extra 700 to 800 patients with fractures each day presenting to Dutch EDs during a period with snow and ice. This massive increase in this specific type of patients gives this epidemic the character of a “major incident” [2]. In contrast to most other major incidents, snow- and ice-related injuries are reasonably predictable [3] and the total ED workload does not seem to increase. This makes management of such an incident easier than management of other major incidents. However, the shift of workload towards traumatology is substantial and hospitals have to be prepared for this patient load during a period with snow and ice. On a community level, prevention of injuries is important, particularly in accidents that occur on a large scale and that have a predictable cause, such as fractures sustained by snow and ice. Optimization of preventive measures, such as prompt de-icing of footpaths and bicycle lanes, could probably reduce the number of fractures sustained during a period with snow and ice. Further research could explore the effect of optimization of preventive measures on fracture prevalence during a period with snow and ice.

### Limitations

This study has several limitations. Firstly, the cross sectional design with its limitations: a causal relationship cannot be determined. Other factors could be involved in the increase in fractures during the snow and ice period.

Secondly, our information about the injury mechanism was limited to the information available in the medical chart and therefore dependant on the documentation by the treating physician and nurse. Exact information about the accident was not always available. To give good advice for any useful preventive measures more information is needed.

Finally it should be noted that the current study only involved patients in the two locations of a hospital in the west of the Netherlands and that extrapolations from the current study should be interpreted with some reservations.

## Conclusions

The number of fractures sustained during the snow and ice period more than doubled as compared to the control period, this is entirely a result of fractures sustained outside. The total number of ED visits did not increase during the snow and ice period, nor did the length of ED stay.

In contrast to other studies outside the Netherlands, it was not the elderly, but the middle-aged who were most affected by the slippery conditions.

## Competing interests

The authors declare no competing interests.

## Authors’ contributions

CvdB had full access to all of the data in the study and takes responsibility for the integrity of the data and the accuracy of the data analysis. Study concept & design: CvdL, CvdB and NvdL. Acquisition of the data: CvdL and CvdB. Analysis and interpretation of data: CvdB and CvdL. Drafting of the manuscript: CvdB, CvdL and NvdL. Critical revision of the manuscript for important intellectual content: SR. All authors read and approved the final manuscript.
